# Bradykinin, as a Reprogramming Factor, Induces Transdifferentiation of Brain Astrocytes into Neuron-like Cells

**DOI:** 10.3390/biomedicines9080923

**Published:** 2021-07-30

**Authors:** Tsong-Hai Lee, Pei-Shan Liu, Su-Jane Wang, Ming-Ming Tsai, Velayuthaprabhu Shanmugam, Hsi-Lung Hsieh

**Affiliations:** 1 Stroke Center and Stroke Section, Department of Neurology, Chang Gung Memorial Hospital, College of Medicine, Chang Gung University, Taoyuan City 33305, Taiwan; thlee@cgmh.org.tw; 2 Department of Microbiology, Soochow University, Taipei City 11102, Taiwan; psliu@scu.edu.tw; 3 School of Medicine, Fu Jen Catholic University, New Taipei City 24205, Taiwan; 049700@mail.fju.edu.tw; 4 Research Center for Chinese Herbal Medicine, Department of Nursing, Division of Basic Medical Sciences, Graduate Institute of Health Industry Technology, Chang Gung University of Science and Technology, Taoyuan City 33303, Taiwan; mmtsai@mail.cgust.edu.tw; 5 Department of Biotechnology, Bharathiar University, Coimbatore 641046, India; velayuthaprabhu@buc.edu.in; 6 Department of Neurology, Chang Gung Memorial Hospital, Taoyuan City 33305, Taiwan

**Keywords:** bradykinin, reprogramming factor, brain astrocytes, transdifferentiation, matrix metalloproteinase-9

## Abstract

Kinins are endogenous, biologically active peptides released into the plasma and tissues via the kallikrein-kinin system in several pathophysiological events. Among kinins, bradykinin (BK) is widely distributed in the periphery and brain. Several studies on the neuro-modulatory actions of BK by the B_2_BK receptor (B_2_BKR) indicate that this neuropeptide also functions during neural fate determination. Previously, BK has been shown to induce differentiation of nerve-related stem cells into neuron cells, but the response in mature brain astrocytes is unknown. Herein, we used rat brain astrocyte (RBA) to investigate the effect of BK on cell transdifferentiation into a neuron-like cell morphology. Moreover, the signaling mechanisms were explored by zymographic, RT-PCR, Western blot, and immunofluorescence staining analyses. We first observed that BK induced RBA transdifferentiation into neuron-like cells. Subsequently, we demonstrated that BK-induced RBA transdifferentiation is mediated through B_2_BKR, PKC-δ, ERK1/2, and MMP-9. Finally, we found that BK downregulated the astrocytic marker glial fibrillary acidic protein (GFAP) and upregulated the neuronal marker neuron-specific enolase (NSE) via the B_2_BKR/PKC-δ/ERK pathway in the event. Therefore, BK may be a reprogramming factor promoting brain astrocytic transdifferentiation into a neuron-like cell, including downregulation of GFAP and upregulation of NSE and MMP-9 via the B_2_BKR/PKC-δ/ERK cascade. Here, we also confirmed the transdifferentiative event by observing the upregulated neuronal nuclear protein (NeuN). However, the electrophysiological properties of the cells after BK treatment should be investigated in the future to confirm their phenotype.

## 1. Introduction

Recent studies in stem cell research have indicated that certain mammalian stem cells, even from adults, might be more plastic than previously thought in that they maintain the ability for multi-lineage cell differentiation and may turn into cells of unrelated lineages in response to environmental cues [1]. In the central nervous system (CNS), neuronal differentiation of stem cells normally results from a gradually progressive restriction in developmental potential and is regulated by specific and temporally precise genetic events [2]. This gradually progressive neural induction and neuronal differentiation have been demonstrated in vitro in embryonic stem (ES) cells, adult neural stem cells, or bone marrow stromal cells (BMSC) [2]. Specific molecular control mechanisms determine the differentiation of totipotent ES cells into neural stem cells that can undergo self-renewal and generate more restricted precursors in response to different factors. Eventually, restricted precursors can differentiate into all cells within the nervous system, including neurons and glia [2,3]. However, several lines of evidence suggest that differentiation may not be entirely a one-way street since it has been shown that tissue-specific stem cells, intermediate precursors, and even fully differentiated postmitotic cells can be induced to alter their phenotypic profiles [4]. Here, we explored whether matured brain astrocytes have the potential to be transdifferentiated into neurons.

Astroglial cells constitute nearly 40% of the total CNS cell population in the adult human brain, which exert a wide range of functions including the guidance of the development and migration of neurons during brain development, participating in the immune and repairing responses to brain injury and diseases [5,6]. Moreover, several lines of evidence have indicated that astrocytes are involved in the regulation of neurogenesis in both intact adult brains and after injury [7,8]. Recent reports have shown that astrocytes, major components of the adult neurogenic niches, are evolving as important regulators of neurogenesis, by controlling NSC proliferation, fate choice, and differentiation of the progeny [9]. These studies indicated that brain astrocytes play a critical role in neurogenic niches. Understanding, on a molecular and cellular level, what factors are able to reprogram astrocytic fate decisions and induce its transdifferentiation into neurons will pave the way for new therapeutic strategies using the astrocytic potential for brain repair.

The characteristic morphological and physiological properties of a neuron are the outcomes of a developmental program involving interaction between the intrinsic properties of the developing neuron and extrinsic cues provided by the environment. The importance of the extrinsic factors in molding a developing neuron into its adult form is manifested in a variety of ways [10]. Among these extrinsic factors, kinins, including bradykinin (BK), may play a role in neural differentiation. BK is an endogenous biologically active peptide released into the plasma and tissues via the kallikrein-kinin system in several pathophysiological events, including infection, tissue trauma, inflammation, and pain [11,12]. BK has been shown to be widely distributed not only in the periphery but also in the brain [13]. Several studies on the developmental and neuromodulatory actions of BK by the B2 BK receptor (B2BKR), a G protein-coupled receptor, indicating that this neuropeptide also functions during neural fate determination [14,15,16]. These studies implied that BK may be a potential factor in influencing neuronal development and differentiation.

Matrix metalloproteinases (MMPs) are a large family of zinc-dependent endopeptidases which is a crucial molecule for the turnover of extracellular matrix (ECM) and pathophysiological processes [17]. In the CNS, MMPs have been demonstrated to participate in morphogenesis, developmental remodeling, wounding healing, and neurite outgrowth [17,18,19]. MMP-9 plays a key physiological role in neuronal precursor migration and apoptosis in the developing cerebellum, and in the temporal regulation of the cerebellar microenvironment [20]. Moreover, there is increasing in vitro evidence for the involvement of MMPs, MMP-9 especially, in neurite elongation and axonal guidance [21,22]. Several studies have indicated that upregulation of MMP-9 may contribute to the pathogenic process of brain diseases by several brain injuries [17]. Previous studies have shown that several proinflammatory mediators including BK can induce MMP-9 expression and MMP-9-related functions in brain astrocytes [23,24,25]. These reports implicated that MMP-9 may play a critical role in brain development, plasticity, and repair, and this has aroused our interest to investigate the effect of BK-induced MMP-9 expression on regulating astrocytic transdifferentiation into neuron cells.

Based on this background and our previous studies in the brain astrocytes [25], the experiments were performed to reveal the effects and molecular mechanisms of BK on reprogramming brain astrocytic differentiation into neuron-like cells. In the study, we found that BK may be a reprogramming factor that can switch astrocytes into neuron-like cells. Moreover, BK-stimulated B2BKR-mediated activation of PKC-δ and ERK1/2 cascade contribute to the astrocytic transdifferentiation events, including the downregulation of GFAP and upregulation of NSE and MMP-9 in these cells. Finally, it is possible that understanding the potential of brain astrocytic transdifferentiation in vitro toward a neural lineage will provide insights into the possible use of these cells, or reprogramming factors like BK associated with their transdifferentiation, in therapeutic approaches for a variety of CNS injuries and neurological disorders.

## 2. Materials and Methods

### 2.1. Materials

Dulbecco’s modified Eagle’s medium (DMEM)/F-12 medium, fetal bovine serum (FBS), and TRIzol were from Invitrogen (Carlsbad, CA, USA). Hybond C membrane and enhanced chemiluminescence (ECL) Western blot detection system were from GE Healthcare Biosciences (Buckinghamshire, UK). PhosphoPlus ERK1/2 (Thr^202^/Tyr^204^) antibody (Cat. #4370) was from Cell Signaling (Danvers, MA, USA). ERK2 antibody (Cat. #sc-154) was from Santa Cruz (Santa Cruz, CA, USA). Anti-glyceraldehyde-3-phosphate dehydrogenase (GAPDH) antibody (Cat. #4699–9555) was from Biogenesis (Bournemouth, UK). Glial fibrillary acidic protein (GFAP) antibody (Cat. #Z0334) was from DAKO (Carpinteria, CA, USA). Neuron-specific enolase (NSE) (Cat. #ab79757) and Neuronal nuclear protein (NeuN) (Cat. #ab177487) antibodies were from Abcam (Waltham, MA, USA). D-Arg-[Hyp^3^, Thi^5^, D-Tic^7^, Oic^8^]-BK (Hoe140), GM6001, rottlerin, and PD98059 were from Biomol (Plymouth Meeting, PA, USA). The bicinchoninic acid (BCA) protein assay reagent was from Pierce (Rockford, IL, USA). Bradykinin (BK), phorbol 12-myristate 13-acetate (PMA), enzymes, and other chemicals were from Sigma (St. Louis, MO, USA).

### 2.2. Cell Cultures and Treatments

The rat brain astrocytic cell line (RBA, CTX TNA2) was purchased from BCRC (Hsinchu, Taiwan) and used throughout this study. Cells were plated onto 12-well culture plates (1.5 × 10^5^ cells/well) and made quiescent at confluence by incubation in serum-free DMEM/F-12 for 24 h, and then incubated with BK (10 nM) at 37 °C for the indicated time intervals. When the inhibitors were used, cells were pretreated with the inhibitor for 1 h before exposure to BK (10 nM). Treatment of RBA with BK or these inhibitors alone had no significant effect on cell viability determined by an XTT assay (data not shown).

### 2.3. Cell Morphological Assay

RBA cells were cultured to confluence in 6-well plates and starved with serum-free DMEM/F-12 medium for 24 h. BK (1 nM) was added to each well as indicated times after pretreatment of inhibitors for 1 h. Images were observed and taken at 0 and 24 h with a digital camera and a microscope (Olympus, Tokyo, Japan). These resulting five phase images (the length of neurite fiber of RBA) for each point were quantified and the statistics were obtained for each experimental condition. The data presented are generated from four separate assays.

### 2.4. MMP Gelatin Zymography

Growth-arrested cells were incubated with BK for the indicated time intervals. After treatment, the cultured media were collected and analyzed by gelatin zymography [24]. The gelatinolytic activity was manifested as horizontal white bands on a blue background. Because cleaved MMPs were not reliably detectable, only pro-form zymogens were quantified.

### 2.5. Preparation of Cell Extracts and Western Blot Analysis

Growth-arrested cells were incubated with BK (10 nM) at 37 °C for the indicated time intervals. The cells were washed with ice-cold PBS, scraped, and collected by centrifugation at 45,000× *g* for 1 h at 4 °C to yield the whole-cell extract, as previously described [24]. Samples were analyzed by Western blot, transferred to a nitrocellulose membrane, and then incubated overnight using an anti-phospho-ERK1/2, ERK2, GFAP, NSE, or GAPDH antibody (1:1000). Membranes were washed four times with TTBS for 5 min each, incubated with a 1:2000 dilution of anti-rabbit horseradish peroxidase antibody for 1 h. The immunoreactive bands were detected by ECL reagents and captured by a UVP BioSpectrum 500 Imaging System (Upland, CA, USA). The image densitometry analysis was quantified by an UN-SCAN-IT gel 6.1 software (Orem, UT, USA).

### 2.6. Total RNA Extraction and Reverse Transcription-PCR Analysis

Total RNA was extracted from RBA cells [24]. The cDNA obtained from 0.5 μg total RNA was used as a template for PCR amplification. Oligonucleotide primers were designed on the basis of Genbank entries for rat GFAP and β-actin. The primers were: GFAP (Fwd: GAAGCAGGGCAAGATGGAGC, Rev: GCTGTTCCAGGAAGCGGCAAT), β-actin (Fwd: GAACCCTAAGGCCAACCGTG, Rev: TGGCATAGAGGTCTTTACGG). The amplification was performed in 30 cycles at 55 °C, 30 s; 72 °C, 1 min; 94 °C, 30 s. PCR fragments were analyzed on 2% agarose 1X TAE gel containing ethidium bromide and their size was compared with a molecular weight marker. Amplification of β-actin, a relatively invariant internal reference RNA, was performed in parallel, and cDNA amounts were standardized to equivalent β-actin mRNA levels. The image densitometry analysis was quantified by an UN-SCAN-IT gel 6.1 software (Orem, UT, USA).

### 2.7. Immunofluorescence Staining

Growth-arrested cells were treated with 10 nM BK for 24 h, washed twice with ice-cold PBS, fixed with 4% (*w*/*v*) paraformaldehyde in PBS for 30 min, and then permeabilized with 0.3% Triton X-100 in PBS for 15 min. The staining was performed by incubating with 10% normal goat serum in PBS for 30 min, followed by incubating with an anti-GFAP, anti-NSE, or anti-NeuN polyclonal antibody (1:200 dilution) for 1 h in PBS with 1% BSA, washing three times with PBS, incubating for 1 h with fluorescein isothiocyanate-conjugated goat anti-rabbit antibody (1:200 dilution) in PBS with 1% BSA, washing three times with PBS, and finally mounting with aqueous mounting medium. The images were observed under a fluorescence microscope (Axiovert 200M; Zeiss, Göttingen Germany).

### 2.8. Statistical Analysis of Data

All data were estimated using the GraphPad Prism 8 (GraphPad, San Diego, CA, USA). Quantitative data were analyzed by one-way ANOVA followed by Tukey’s honestly significant difference tests between individual groups. Data were expressed as the mean ± SEM. A value of *p* < 0.05 was considered significant.

## 3. Results

### 3.1. The Bradykinin (BK) Induces Cell Morphological Change of Brain Astrocyte into Neuron-like Cell through a B_2_BK Receptor

**Figure 1:**
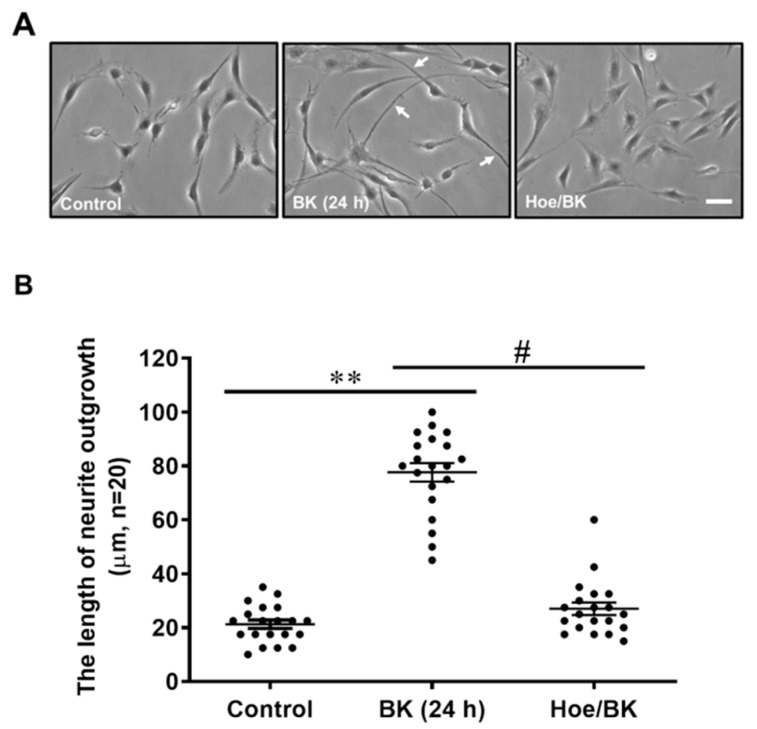
Bradykinin (BK) induces cell morphological change of brain astrocyte into neuron-like cells through a B_2_ BK receptor. RBA cells were pretreated with Hoe140 (10 μM) for 1 h and then incubated without or with BK (10 nM) for 24 h. Representative phase contrast images were obtained (**A**, scale bar = 10 μm) and shown for 24 h (**A**) and the length of neurite fiber outgrowth at 24 h was quantified ((**B**), n = 20). Data are expressed as the mean ± SEM of at least three independent experiments. ** *p* < 0.01, as compared with control. ^#^ *p* < 0.05, as compared with BK alone.

First, we were surprised to find that treatment of rat brain astrocytes (RBA) with BK (10 nM) significantly changed the RBA morphology into neuron-like cells during the period of observation (Figure 1A). As shown in Figure 1A (middle image), BK induced RBA cell neurite fiber outgrowth like neuronal axon at 24 h, suggesting that BK might be a reprogramming factor that can directly reprogram RBA into neuronal-like cells. Therefore, we determine the role of BK in the event. Previous reports indicated that BK interacts with two BK receptor subtypes, which have been classified as B_1_ and B_2_ BK receptors [26]. Astrocytes are known to express B_2_-type BK receptors [27]. Our previous studies have demonstrated that RBA expresses the B_2_BK receptor by a radioligand [^3^H]-BK binding assay, immunofluorescence staining, and Western blotting analysis [23,28]. Here, to determine whether BK-induced RBA morphological change into neuron-like cells is mediated through the B_2_BK receptor, a B_2_BK receptor antagonist Hoe140 was used. Pretreatment of RBA with Hoe140 (10 μM) markedly blocked BK-induced RBA morphological change into neuron-like cells (Figure 1A, right image). The length of neurite fiber outgrowth was quantified and shown in Figure 1B, suggesting that BK can directly induce brain astrocyte morphological change into a neuron-like cell via a B_2_BK receptor-dependent manner.

### 3.2. BK-Induced RBA Morphological Change into Neuron-like Cell Is Mediated through MMP-Dependent Manner

**Figure 2:**
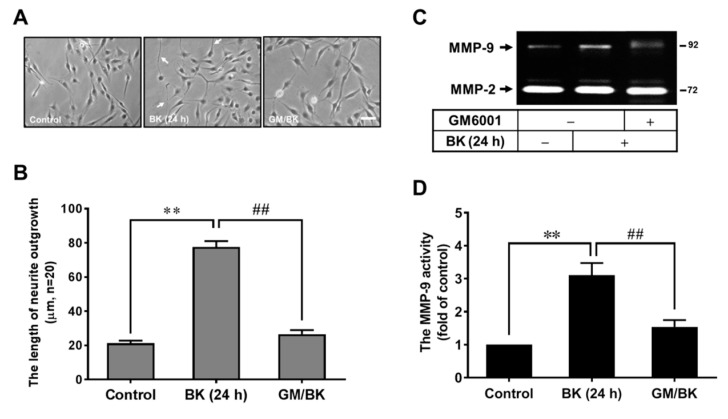
BK-induced RBA morphological change into neuron-like cells is mediated through an MMP-dependent manner. RBA cells were pretreated with GM6001 (10 μM) for 1 h and then incubated with or without BK (10 nM) for 24 h. Representative phase-contrast images were obtained ((**A**), scale bar = 20 μm) and shown for 24 h (**A**) and the length of neurite fiber outgrowth at 24 were quantified ((**B**), n = 20). Cells were pretreated with or without GM6001 (10 μM) for 1 h before exposure to 10 nM BK for 24 h. The conditioned media were collected and analyzed by gelatin zymography (**C**) and quantified (**D**). The image represents one of at least three individual experiments. Data are expressed as the mean ± SEM of at least three independent experiments. ** *p* < 0.01, as compared with control. ^##^ *p* < 0.01, as compared with BK alone.

In the CNS, MMPs are implicated in several physiological events, including morphogenesis and neurite outgrowth [17]. Previously, we have demonstrated that BK upregulates MMP-9 expression, but not MMP-2, in RBA [23]. Here, to investigate whether MMP-9 participates in BK-induced RBA morphological change into neuron-like cells, RBA were pretreated with an MMP inhibitor GM6001 for 1 h and then incubated with BK for 24 h. As shown in Figure 2A, pretreatment with GM6001 (10 μM) inhibited BK-induced RBA morphological change into neuron-like cells and the statistical results of the length of neurite fibers are shown in Figure 2B. To confirm the effect of MMPs, MMP-9 especially, on the BK-induced event, the conditioned media were collected and analyzed the MMP expression and activity by gelatin zymography. As our previous report showed that BK induced MMP-9 expression in RBA, the MMP-9 activity was attenuated by pretreatment with GM6001 (Figure 2C). The statistical data of MMP-9 activity by gelatin zymography were shown in Figure 2D. These results suggest that MMPs, MMP-9 especially, might be a critical factor for BK-induced RBA morphological change into neuron-like cells.

### 3.3. Involvement of PKC-δ in BK-Induced RBA Morphological Change into Neuron-like Cell

**Figure 3:**
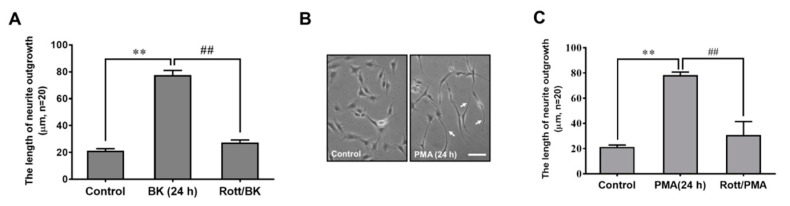
Involvement of PKC-*δ* in BK-induced RBA morphological change into neuron-like cells. (**A**) RBA cells were pretreated with rottlerin (1 μM) for 1 h and then incubated with or without BK (10 nM) for 24 h. (**B**) Cells were treated without (control) or with PMA (1 μM) for 24 h. (**C**) Cells were pretreated with rottlerin (10 μM) for 1 h before exposure to 1 μM PMA for 24 h. The phase-contrast images were obtained ((**B**), scale bar = 20 μm) and the length of neurite fiber outgrowth at 24 h was quantified (**A**,**C**). The image represents one of at least three individual experiments (**B**). Data are expressed as the mean ± SEM of at least three independent experiments. ** *p* < 0.01, as compared with control. ^##^ *p* < 0.01, as compared with BK or PMA alone.

Our previous data have shown that upregulation of several critical proteins like MMP-9 requires PKC-*δ*-mediated activity [24]. Thus, to determine the role of PKC-*δ* in BK-induced RBA morphological change into neuron-like cells, the selective PKC-*δ* inhibitor rottlerin was used. As shown in Figure 3A, pretreatment with rottlerin (1 μM) caused a significant inhibition of BK-induced RBA morphological change into neuron-like cells, suggesting that PKC-*δ* may play a potential role in the event. Additionally, we also used a PKC activator, phorbol 12-myristate 13-acetate (PMA), to confirm the role of PKC-*δ* in the response, RBA were directly treated with PMA (1 μM) for 24 h and then the images were captured and analyzed. The results showed that PMA also induced RBA morphological change into neuron-like cells (Figure 3B). Similarly, pretreatment of cells with rottlerin markedly blocked PMA-induced RBA morphological change into neuron-like cells (Figure 3C). These data demonstrated that PKC-*δ* plays a critical role in BK-induced morphological change of brain astrocytes into neuron-like cells.

### 3.4. The ERK Participates in BK-Induced RBA Morphological Change into Neuron-like Cell

**Figure 4:**
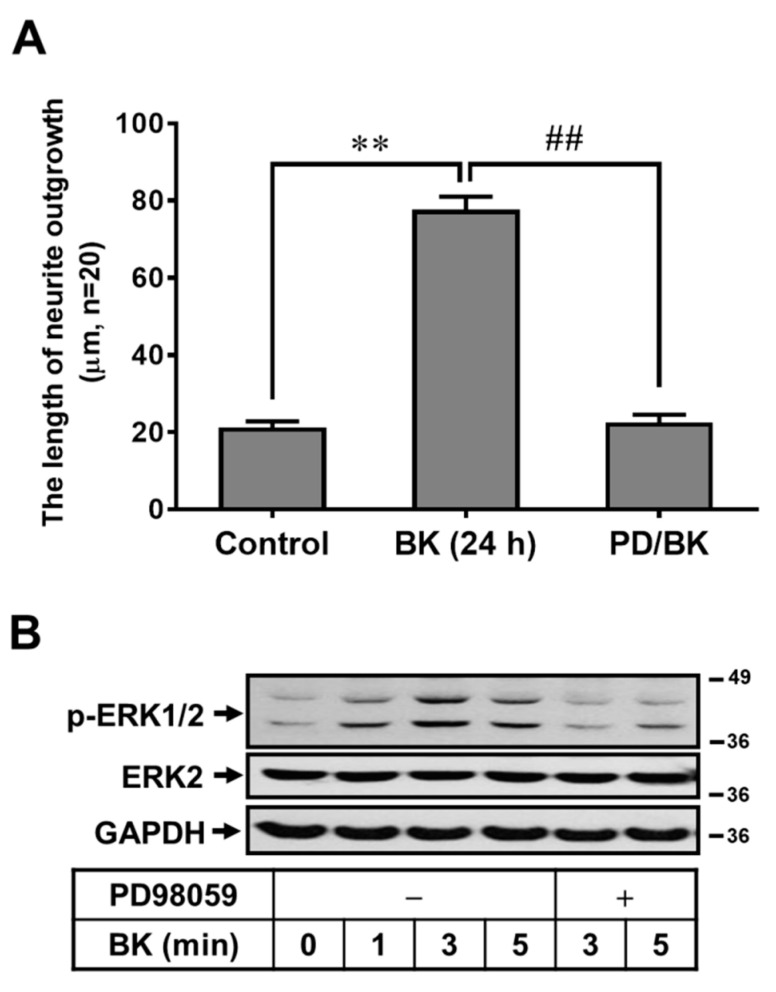
The ERK1/2 participates in BK-induced RBA morphological change into neuron-like cells. (**A**) RBA cells were pretreated with PD98059 (10 μM) for 1 h and then incubated with or without BK (10 nM) for 24 h. The phase-contrast images were obtained and the length of neurite fiber outgrowth at 24 h was quantified. (**B**) Cells were pretreated without or with BK (10 nM) for the indicated times. The whole-cell lysates were subjected to 10% SDS-PAGE and analyzed using an anti-phospho-ERK1/2, anti-ERK2, or anti-GAPDH (as an internal control) antibody, as described under ‘‘Methods’’. The image represents one of at least three individual experiments. Data are expressed as the mean ± SEM of at least three independent experiments. ** *p* < 0.01, as compared with control. ^##^ *p* < 0.01, as compared with BK alone.

Activation of MAPKs by various stimuli could affect brain cell functions [25,29]. Previous reports have shown that ERK1/2 is critical for the upregulation of MMP-9 in brain astrocytes [24]. Thus, to determine whether ERK1/2 participates in BK-induced RBA morphological change into neuron-like cells, cells were pretreated with PD98059 (10 μM) for 1 h and then incubated with BK (10 nM) for 24 h. As shown in Figure 4A, pretreatment with PD98059 blocked BK-induced RBA morphological change into neuron-like cells, suggesting that ERK1/2 may be involved in the BK-induced RBA response. We further demonstrated that BK stimulated, time-dependently, ERK1/2 phosphorylation with a maximal response within 3 min by Western blot (Figure 4B). Moreover, cells were pretreated with PD98059 and then incubated with BK (10 nM) for the indicated time intervals. The results showed that pretreatment with PD98059 (10 μM) significantly attenuated BK-stimulated ERK1/2 phosphorylation during the period of observation (Figure 4B). These results suggested that BK-induced morphological change of brain astrocyte into neuron-like cells is mediated through the ERK1/2-dependent pathway.

### 3.5. BK Downregulates Astrocytic Marker GFAP Expression in RBA

**Figure 5:**
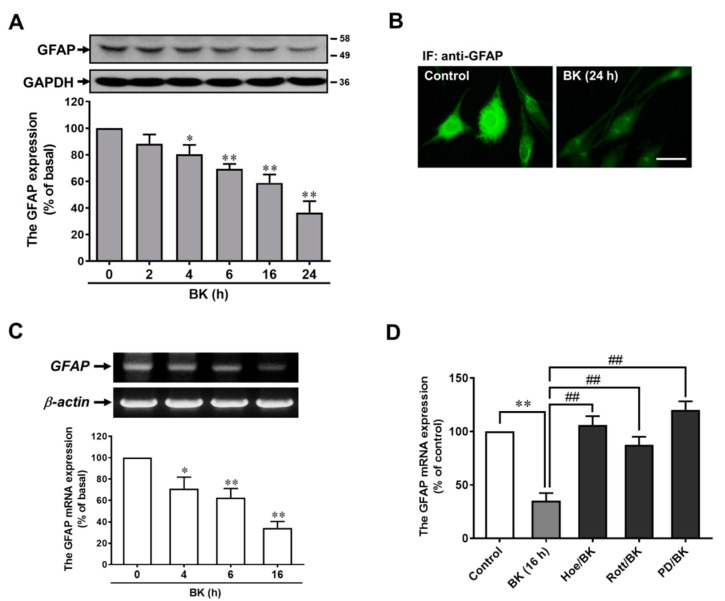
BK downregulates astrocytic marker GFAP expression in RBA cells. (**A**) Time dependence of BK-induced downregulation of GFAP protein expression. RBA cells were treated with 10 nM BK for the indicated times. The whole-cell lysates were analyzed by Western blot using an anti-GFAP or anti-GAPDH antibody. (**B**) The downregulation of GFAP by BK was confirmed by immunofluorescence in RBA cells. Cells were treated without (control) or with BK (10 nM) for 24 h and then labeled using an anti-GFAP antibody and a FITC-conjugated secondary antibody. Individual cells were imaged (scale bar = 20 μm) as described in ‘‘Methods’’. (**C**) Time dependence of BK-downregulated GFAP mRNA expression. RBA cells were treated with 10 nM BK for the indicated times. (**D**) Cells were pretreated with Hoe140 (Hoe, 10 μM), rottlerin (Rott, 1 μM), PD98059 (PD, 10 μM) for 1 h and then incubated with BK (10 nM) for 16 h. The total RNA was extracted and analyzed by RT-PCR as described under ‘‘Methods’’. Data are expressed as the mean ± SEM of at least three independent experiments. * *p* < 0.05, ** *p* < 0.01, as compared with control. ^##^ *p* < 0.01, as compared with BK alone. The image represents one of at least three individual experiments.

The GFAP is a marker for astrocytes, known to be induced upon brain damage or during CNS degeneration, and to be more highly expressed in the aged brain [30,31]. Moreover, GFAP has been shown to participate in astrocytic functions, which are important during brain development, regeneration, synaptic plasticity, and reactive gliosis [30,31]. Thus, we further explored whether the expression of GFAP is affected in the process of BK-induced RBA morphological change into neuron-like cells. As shown in Figure 5A, the Western blotting data showed that the expression of GFAP protein was time-dependently downregulated by treatment with BK. There was a significant decrease within 4 h (reduced to ~80.25% of basal), which sustained up to 24 h (reduced to ~36.5% of basal). BK-downregulated GFAP expression was further supported by the results obtained using immunofluorescence staining against a GFAP antibody. The result showed that BK indeed caused downregulation of GFAP in RBA (Figure 5B). To further examine whether the downregulation of GFAP protein by BK results from the decrease of GFAP mRNA expression, the RT-PCR analysis was performed. As shown in Figure 5C, BK time-dependently downregulated GFAP mRNA expression in RBA. There was a significant decrease in GFAP mRNA within 4 h, and this was sustained over 16 h. These data demonstrated that BK downregulated GFAP expression at the transcriptional level. Next, we determined the involvement of the B_2_BKR/PKC-δ/ERK pathway in BK-downregulated GFAP mRNA expression by using various specific inhibitors. The RBA was pretreated with Hoe140 (10 μM), rottlerin (1 μM), or PD98059 (30 μM) for 1 h and then treated with BK (10 nM) for 16 h. The total RNA was extracted and analyzed by RT-PCR. These results showed that pretreatment with these inhibitors significantly prevented BK-downregulated GFAP mRNA expression (Figure 5D), suggesting that BK downregulated GFAP gene expression via the B_2_BKR/PKC-δ/ERK cascade in RBA.

### 3.6. BK Induces Upregulation of Neuronal Marker NSE in Transdifferentiation of RBA

**Figure 6:**
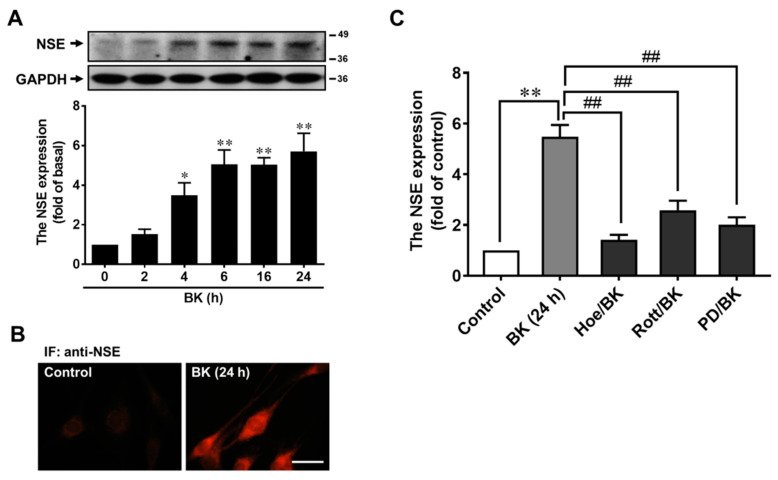
BK upregulates the neuronal marker NSE expression in RBA cells. (**A**) Time dependence of BK-induced upregulation of NSE protein expression. Cells were treated with 10 nM BK for the indicated times. (**B**) The upregulation of NSE by BK was confirmed by immunofluorescence in RBA cells. Cells were incubated without (control) or with BK (10 nM) for 24 h and then labeled using an anti-NSE antibody and a FITC-conjugated secondary antibody. Individual cells were imaged (scale bar = 20 μm) as described in ‘‘Methods’’. (**C**) Cells were pretreated with Hoe140 (Hoe, 10 μM), rottlerin (Rott, 1 μM), PD98059 (PD, 10 μM) for 1 h and then incubated with BK (10 nM) for 24 h. The whole-cell lysates were analyzed by Western blot using an anti-NSE or anti-GAPDH antibody as described under ‘‘Methods’’. Data are expressed as the mean ± SEM of at least three independent experiments. * *p* < 0.05, ** *p* < 0.01, as compared with control. ^##^ *p* < 0.01, as compared with BK alone. The image represents one of at least three individual experiments.

Based on these results above, we speculated that BK might induce RBA to transdifferentiate into neuronal cells. To examine the speculation, we first detected the expression of NSE, which is widely used as a neuron marker, in BK-induced RBA by Western blot. The results showed that BK induced NSE expression in a time-dependent manner (Figure 6A), a marked increase within 4 h and sustained up to 24 h. In addition, BK-induced NSE expression was further confirmed by immunofluorescence staining. The IF image data showed that BK induced NSE expression in RBA (Figure 6B). Next, to determine whether the B_2_BKR/PKC-δ/ERK cascade is involved in BK-induced NSE expression, these specific inhibitors were used. As shown in Figure 6C, pretreatment with Hoe140 (10 μM), rottlerin (1 μM), or PD98059 (30 μM) significantly inhibited BK-induced NSE expression, indicating that BK-induced NSE expression is mediated through the B_2_BKR-dependent activation of PKC-δ/ERK pathway in RBA.

## 4. Discussion

Astrocytes, a glial cell, are broadly distributed throughout the CNS. Moreover, it is well known that astrocytes have multiple effects on CNS physiological and pathological processes, including maintaining homeostasis, providing neurotrophins, and regulating neural signal transmission [5,32,33,34]. Increasing reports have shown that astrocytes may also be neural progenitor cells and contribute to adult neurogenesis or neuroregeneration [9,34]. In pathological conditions, glial cells could be reactivated to proliferate and differentiate [33,35]. When cultured in vitro, they could form neurospheres that possess the ability to differentiate into neurons. Additionally, forced expression of exogenous genes in glial cells including astrocytes can successfully reprogram them into neurons, which may also be suggestive of their progenitor cell features [33,34]. Here, we found that BK-induced brain astrocytes (RBA) exhibited a neuron-like phenotype, suggesting that BK may be a reprogramming factor in switching transdifferentiation of brain astrocytes into neuron-like cells. Moreover, BK stimulated B_2_BKR-mediated activation of PKC-δ and ERK1/2 cascade to contribute to the astrocytic transdifferentiating events, including downregulation of GFAP and upregulation of NSE and MMP-9 in RBA. Finally, it is possible that understanding the transdifferentiation potential of brain astrocytes in vitro toward a neural lineage will provide insights into the possible use of these cells, or reprogramming factors like BK associated with their transdifferentiation, in therapeutic approaches for a variety of CNS injuries and neurological disorders.

BK is the biologically active peptide of the kallikrein–kinin system that interacts with two BK receptor subtypes, including B_1_- and B_2_-type. In the CNS, astrocytes are known to express B_2_BK receptors, and this type is found only on astrocytes type-1 [27,33]. A previous study has found that BK causes PC-12 cells to extend neurites and BK potentiates the neurite-extending effect of NGF, an action which is attenuated by a BK antagonist [16]. Moreover, BK treatment simultaneously induces neuronal enrichment (indicating that BK contributes to neurogenesis) and reduced proliferation rates during in vitro differentiation of rat embryonic telencephalon neural precursor cells [14,15]. These studies suggest that the neuropeptide BK also functions during neuronal development and neuromodulation. Here, we are the first study to report that BK induces RBA morphological change into neuron-like cells such as neurite-like extension, suggesting that BK may play a switching role in the transdifferentiation of RBA into neuron-like cells (Figure 1). Next, we demonstrated that BK-induced the RBA morphological change (i.e., fibrous outgrowth) into neuron-like cells is mediated through a B_2_BKR-dependent manner. The result is consistent with a previous report that indicated that BK-induced B_2_BK receptor-mediated signals provide a switch for neural fate determination [15]. Therefore, we suggest that the neurogenic properties of BK described herein may open novel avenues for the therapy of neurodevelopmental and neurodegenerative diseases.

In the CNS, MMPs contribute to a wide range of biological activities, including morphogenesis, developmental remodeling, wounding healing, and neurite outgrowth [17,18,19]. Among MMPs, regulation of MMP-9 plays a critical role in physiological and pathological events, including neuronal precursor migration and apoptosis in the developing cerebellum [20] or in pathogenic processes of brain diseases [17,18,19]. Moreover, in vitro studies have shown that MMP-9 is involved in neurite elongation and axonal guidance [21,22]. These results suggest that the MMP-9-mediated neuronal development by some factors may provide a therapeutic strategy to neural repair of brain injury and neuronal regeneration of neurodegenerative diseases. Moreover, BK, and related peptides, are simultaneously produced and released following brain injury [25]. Previous studies have demonstrated that BK induces MMP-9 expression and then changes astrocytic functions [36]. Therefore, we explored the effect of MMP-9 on BK-induced RBA morphological change into neuron-like cells. The results showed that GM6001, a broad-spectrum MMP inhibitor, significantly inhibited BK-induced RBA cell fibrous outgrowth and MMP-9 expression (Figure 2), indicating that MMPs (i.e., MMP-9) participate in BK-induced RBA morphological changed events.

During postnatal development of the brain, PKC isozymes such as PKC-δ are expressed in different brain regions [37]. In the cerebella of neonatal rats, the immunoreactivity of PKC-δ is moderate to strong in radial glia, Bergmann fibers, and astrocytes but is absent in neurons [38]. A transient occurrence of PKC-δ in glia and later appearance in selective groups of neurons strongly support a significant role for this enzyme in signal transduction [38]. Our previous data have demonstrated that activation of PKC-δ by BK contributes to upregulation of MMP-9 in brain astrocytes which may change astrocytic functions [24]. Thus, we investigated the role of PKC-δ in BK-induced RBA morphological events. The results showed that BK-induced RBA morphological change into neuron-like cells was blocked by a PKC-δ specific inhibitor rottlerin (Figure 3A). We further used a PKC activator PMA to confirm this result. As expected, PMA can induce RBA morphological change into neuron-like cells which was inhibited by pretreatment with rottlerin (Figure 3B,C). This result was consistent with the result of the BK treatment. These data demonstrated that PKC-δ is essential for BK-induced RBA morphological change into neuron-like cells.

The MAPKs are important signals for the regulation of many cellular processes, such as cell growth, proliferation, differentiation, and apoptosis [39]. In mammalian cells, three major groups of MAPKs have been identified: extracellular signal-regulated kinase (ERK), c-jun N-terminal kinase (JNK), and p38 MAPK. In the CNS, the ERK1/2 is abundant and is activated during various physiological and pathological events including synaptic plasticity, brain development, repair, and memory formation [40,41]. Moreover, a report indicated that ERK1/2 may be the most attractive signal among protein kinases that mediate morphological differentiation in neurons [41]. Previously, we have demonstrated that ERK1/2 is required for upregulation of MMP-9 by BK in brain astrocytes [24,36]. Here, we found that activation of ERK1/2 participated in BK-induced RBA morphological change into neuron-like cells (Figure 4), suggesting that ERK1/2 may be a critical molecule for BK-induced RBA morphological changed event. The results are similar to a report that showed that ERK1/2 is involved in the regulation of differentiation of retinoblastoma cells [42]. Moreover, another study indicated that BK promotes neuron-generating division of neural progenitor cells through ERK activation [43].

The GFAP is a main intermediate filament protein in mature astrocytes throughout the nervous system. Previous reports indicated that GFAP plays an important role in the structure and mobility of astrocytes, and GFAP can influence astrocytic functions during homeostasis, development, regeneration, synaptic plasticity, and reactive gliosis [30]. Moreover, GFAP is a highly regulated protein, whose expression is induced by multiple factors such as brain injury and disease [30], and differences in GFAP expression are indicative of different functions of astrocytes. Moreover, GFAP expression changes might alter the astrocytic morphology, which could indirectly affect other cell types and the structure of the brain. Herein, we found that incubation of RBA with BK time-dependently reduced the expression of GFAP mRNA and protein (Figure 5). Moreover, the BK-downregulated GFAP was inhibited by pretreatment with Hoe140, rottlerin, and PD98059, suggesting that BK-reduced GFAP expression is mediated through B_2_BKR, PKC-δ, and ERK1/2 signals in RBA. It is consistent with the study by using a wounding-in-a-dish model [44,45], the results indicated that treatment with antisense GFAP decreased astrocyte fibrosis, reorganized extracellular laminin, and greatly enhanced neurite outgrowth [45,46]. Similarly, neurite outgrowth was enhanced in astrocytes from GFAP-/- mice [47]. These studies indicated that GFAP plays a critical role in neurite outgrowth.

**Figure A1:**
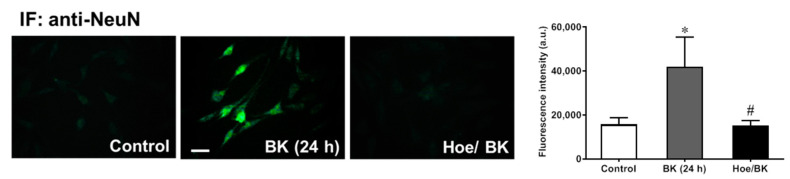
BK induces neuronal nuclear protein (NeuN) expression in RBA. The upregulation of NeuN by BK was confirmed by immunofluorescence in RBA. Cells were pretreated with Hoe140 (Hoe, 10 μM) for 1 h and then incubated with BK (10 nM) for 24 h. After treatment, the cells were labeled using an anti-NeuN antibody and a FITC-conjugated secondary antibody. Individual cells were imaged (scale bar = 20 μm) as described in ‘‘Methods’’. The fluorescence intensity (arbitrary units: a.u.) of NeuN was quantified in these cells. * *p* < 0.05, as compared with control. ^#^ *p* < 0.05, as compared with BK alone.

The NSE is the neuronal form of the glycolytic enzyme enolase which is found almost exclusively in all classes of neurons and cells of neuroendocrine origin [48], and the enzyme levels detected have been used as an index for neuronal differentiation. NSE influences neurotrophic activity and is believed to regulate differentiation and neurite regeneration of neurons via activation of intracellular signaling molecules such as MAPK [48]. These studies indicate that NSE plays a key role during brain development as well as during the repair of injured neurons in the adult CNS. Besides, NSE is also detectable in glial neoplasms and reactive glial cells while undergoing morphological changes [49]. In astrocytes, NSE is expressed but at a lower level than those in cultured neurons [48,49,50]. Furthermore, it has been suggested that glial cells in response to injury may reexpress fetal characteristics of progenitors. This response may represent a reversion to a common origin of glial cells/neurons [50]. Here, we also found that NSE is slightly present in the cultured RBA (Figure 6A), consistent with the study in rat brain type 1 astrocytes [50]. Moreover, BK can induce a time-dependently increase of NSE protein in RBA by Western blot analysis (Figure 6A), and the same result was also observed by immunofluorescent staining of NSE (Figure 6B). Furthermore, BK-induced NSE expression is mediated through B_2_BKR linking to PKC-δ and ERK1/2 signals in RBA (Figure 6C), concluding that BK-induced signaling pathways may provide a switch for astrocytic transdifferentiation into neuron-like cells. Here, we also confirmed the BK-induced astrocytic transdifferentiation into neuron-like cells by observing the expression of the neuronal nuclear protein (NeuN) (Figure A1 of Appendix A). The perspective is consistent with Trujillo et al. indicated that kinin-B_2_ receptor activity determines the differentiation fate of neural stem cells such as neural fate determination [15]. However, a limitation of this study is that in order to clearly determine the cell phenotype after BK treatment, the electrophysiological properties and detailed phenotype of the cells should be examined in the future.

**Figure 7:**
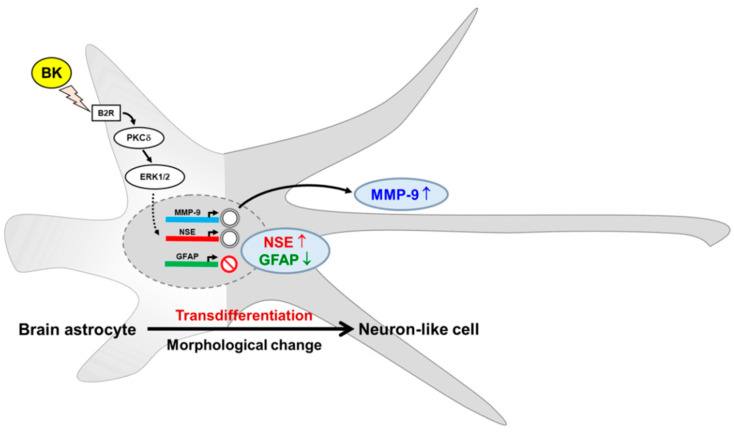
Schematic illustration of the BK-induced signaling pathways linked to reprogram RBA cells into neuron-like cells. Schematic representation of signaling pathways involved in BK-mediated upregulation of MMP-9 and NSE, downregulation of GFAP, and induction of morphological change into neuron-like cells in RBA cells. The binding of BK to its receptor (B_2_BKR) results in the activation of PKC-δ and ERK1/2 cascades. These events of BK-induced RBA cell morphological change into neuron-like cells, including regulation of various proteins, which are mediated through the B_2_BKR/PKC-δ/ERK pathway. Moreover, the upregulation of MMP-9 is critical for BK-induced RBA cell neurite fiber outgrowth in this process.

In conclusion, based on the observations from the literature and our findings, Figure 7 depicts a model for the effect of BK on the induction of RBA morphological change into neuron-like cells. Herein, the data showed that treatment of RBA with BK can induce the cell morphological change (e.g., fibrous outgrowth) into neuron-like cells. BK-induced RBA transdifferentiation is mediated through B_2_BKR-dependent activation of PKC-δ and ERK1/2 signals. Subsequently, BK upregulated MMP-9 and NSE expression, and the GFAP was downregulated at the same time. These findings concerning BK may be as a restarter in switching matured brain astrocytic transdifferentiation into neuron-like cells. Recently, many reports have indicated that the conversion of astrocytes from different brain regions into different functional neurons represents a potential therapeutic approach for replenishing neuronal loss associated with neurodegenerative diseases and brain injury [51,52]. Therefore, the ability to transdifferentiate an easily accessible cell source such as brain astrocytes into a neural lineage could have substantial potential for promoting neural repair in therapeutic approaches for a variety of CNS injuries and neurological disorders.

## Data Availability

The data presented in this study are available in this article.
